# Evolution of ion channels in cetaceans: a natural experiment in the tree of life

**DOI:** 10.1038/s41598-024-66082-1

**Published:** 2024-07-23

**Authors:** Cristóbal Uribe, Mariana F. Nery, Kattina Zavala, Gonzalo A. Mardones, Gonzalo Riadi, Juan C. Opazo

**Affiliations:** 1 Department of Bioinformatics, Program in Sciences Mention Modeling of Chemical and Biological Systems, School of Bioinformatics Engineering, Center for Bioinformatics, Simulation and Modeling, CBSM, Faculty of Engineering, University of Talca, Campus Talca, Talca, Chile; 2 Departamento de Genética, Evolução, Microbiologia e Imunologia, Instituto de Biologia, Universidade Estadual de Campinas-UNICAMP, Cidade Universitária, Campinas, Brazil; 3 Facultad de Medicina y Ciencia, Universidad San Sebastián, Valdivia, Chile; 4 Integrative Biology Group, Valdivia, Chile; 5 Department of Bioinformatics, Center for Bioinformatics, Simulation and Modeling, Faculty of Engineering, CBSM, University of Talca, Talca, Chile; 6 Millennium Nucleus of Ion Channel-Associated Diseases (MiNICAD), Valdivia, Chile

**Keywords:** Na_V_1.5, SCN5A, TTX, PKD1L1, Gene turnover, Tetrodotoxin, Evolution, Molecular evolution

## Abstract

Cetaceans represent a natural experiment within the tree of life in which a lineage changed from terrestrial to aquatic habitats. This shift involved phenotypic modifications, representing an opportunity to explore the genetic bases of phenotypic diversity. Among the different molecular systems that maintain cellular homeostasis, ion channels are crucial for the proper physiological functioning of all living species. This study aims to explore the evolution of ion channels during the evolutionary history of cetaceans. To do so, we created a bioinformatic pipeline to annotate the repertoire of ion channels in the genome of the species included in our sampling. Our main results show that cetaceans have, on average, fewer protein-coding genes and a higher percentage of annotated ion channels than non-cetacean mammals. Signals of positive selection were detected in ion channels related to the heart, locomotion, visual and neurological phenotypes. Interestingly, we predict that the NaV1.5 ion channel of most toothed whales (odontocetes) is sensitive to tetrodotoxin, similar to NaV1.7, given the presence of tyrosine instead of cysteine, in a specific position of the ion channel. Finally, the gene turnover rate of the cetacean crown group is more than three times faster than that of non-cetacean mammals.

## Introduction

Understanding the genetic basis of phenotypic diversity represents a central goal in evolutionary biology. The availability of whole-genome sequences in representative species of different groups represents a unique opportunity to advance this goal. Indeed, the Tree of Life could be seen as a set of natural experiments that help us understand different evolutionary phenomena. For example, lineages have evolved traits of biomedical interest (e.g., cancer resistance), representing an opportunity to understand how the evolutionary process solves problems from which we can potentially gain medical insights^1–6^. Further, during the evolutionary history of vertebrates, colonizations of new habitats have resulted in severe phenotypic transformations, providing an opportunity to understand the genomic basis of diversity^7–11^.

The colonization of the aquatic environment by tetrapods occurred multiple times during their evolutionary history. Among mammals, the cetaceans (whales and dolphins) started transitioning from land to the sea during the Eocene around 50 Mya. A few million years later, the lineage entirely depended on the aquatic environment and diversified and occupied all the seas and many rivers of the world^12^. The successful aquatic colonization from a terrestrial habitat demanded biological transformation due to gravity-related challenges, thermal regimes, a new pathogenic environment, different environmental stimuli that required sensory adaptations, and osmotic regulation, among others^13,14^. Taking advantage of the advancement of genomic tools, these adaptations have been investigated from the molecular perspective to expand further our knowledge about the evolutionary process behind the conquest of an aquatic lifestyle^7–9,15–18^. Interestingly, several studies have reported the loss of genes as a strategy of phenotypic evolution^15,17,19–26^.

Ion channels are integral membrane proteins that allow the passage of ions involved in a diverse repertoire of physiological processes. In the human and mouse genomes, 235 and 231 putative ion channels have been identified, respectively^27^. There is no doubt that ion channels represent a crucial part of the molecular machinery for the correct physiological functioning of living creatures. In fact, amino acid sequence variation has been linked to a wide range of pathological conditions, also called channelopathies^28–30^. Thus, given their pivotal role in different physiological axes, some of which have diverged extensively in cetaceans due to the aquatic transition, it seems interesting to study their evolutionary trend in this mammalian group^31–33^.

This work aims to study the evolution of ion channels during the evolutionary history of cetaceans. To do so, we first created a bioinformatic pipeline to annotate the whole repertoire of ion channels in the genome of the species included in our sampling. After that, we estimated homologous relationships to study the role of positive selection and the variation in gene turnover rate. Our main results show (1) on average, cetaceans possess fewer protein-coding genes and a higher percentage of annotated ion channels than non-cetacean mammals (2) the signal of positive selection was found in ion channels related to heart, locomotion, visual and neurological phenotypes, all characteristics extensively modified in cetaceans, (3) the Na_V_1.5 ion channel of toothed whales (odontocetes), other than species of the genus *Tursiops*, is predicted to be sensitive to the potent neurotoxin tetrodotoxin (TTX), similar to Na_V_1.7, given a replacement of cysteine for a tyrosine, 4) the gene turnover rate of the cetacean crown group is more than three times faster in comparison to non-cetacean mammals.

## Material and methods

### Phylogenetic design, DNA sequences, and ion channel annotation

Our phylogenetic design included 18 mammalian species: seven toothed whales (Odontoceti) (bottlenose dolphin, *Tursiops truncatus*; orca, *Orcinus orca*; beluga, *Delphinapterus leucas*; Yangtze river dolphin, *Lipotes vexillifer*; sperm whale, *Physeter catodon, vaquita, Phocoena sinus, narwhal, Monodon monoceros*), two baleen whales (Mysticeti) (common minke whale, *Balaenoptera acutorostrata*; blue whale, *Balaenaenoptera musculus*), three artiodactyls (hippo, Hippopotamus amphibius, cow, *Bos taurus*; pig, *Sus scrofa*), one carnivore (dog, *Canis familiaris*); one perissodactyl (horse, *Equus caballus*); one chiroptera (microbat, *Myotis lucifugus*); one primate (human, *Homo sapiens*); one rodent (mouse, *Mus musculus*) and one proboscidean (African elephant, *Loxodonta africana*).

**Figure 1:**
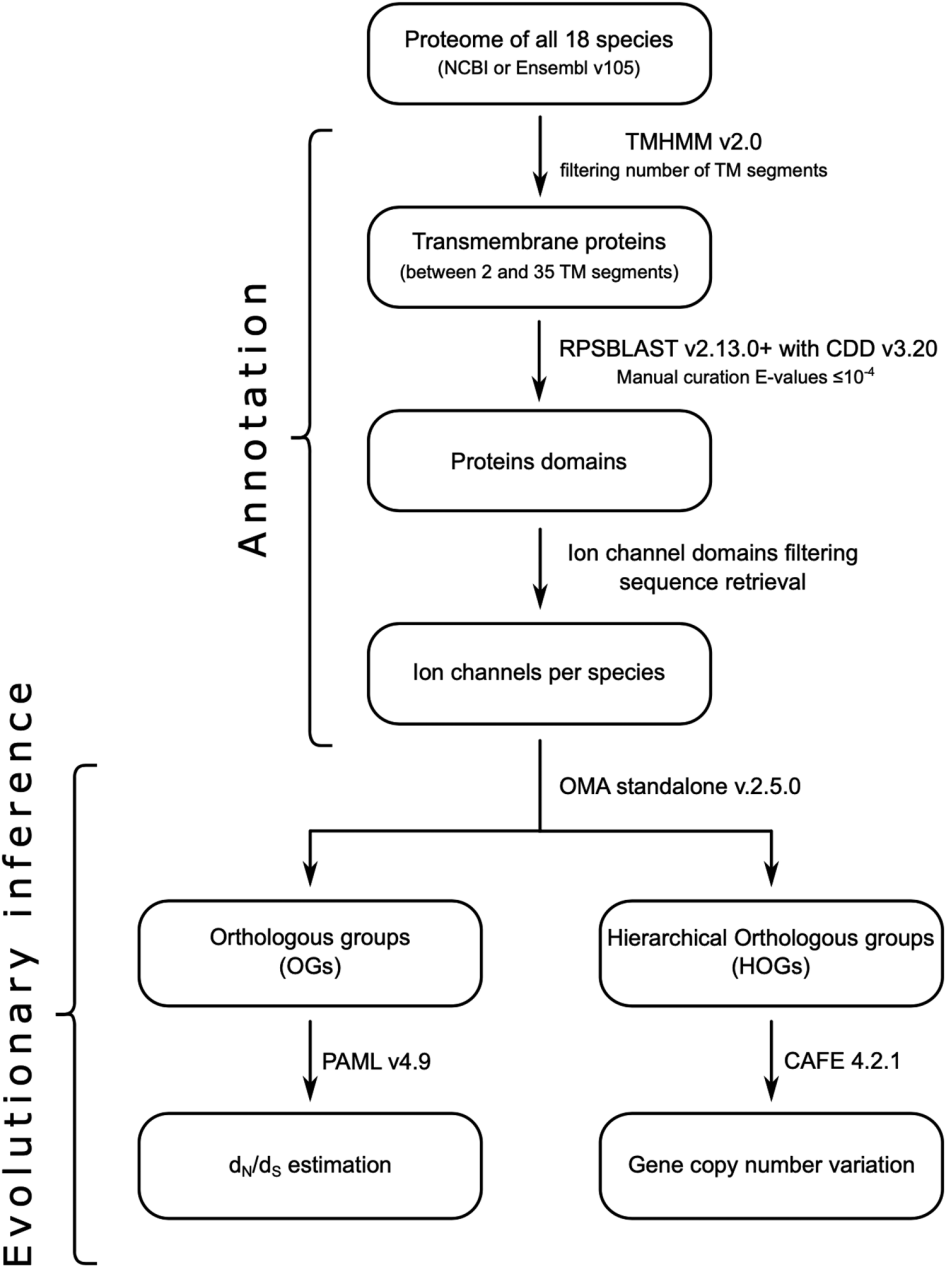
Flow diagram of the methodology used in this work. We divided the pipeline into two main steps, the annotation process, and the evolutionary inference. In the annotation process, we implemented tools like TMHMM^36^ and RPSBlast^37^ to identify the repertoire of ion channels in the proteome of each species. Then, we applied the OMA standalone program to infer orthologous and hierarchical orthologous groups. After that, we used PAML^42^ to estimate the ratio of the rate of non-synonymous (d_N_) and synonymous substitutions (d_S_) (ω = d_N_/d_S_) and CAFE^44^ to study gene copy number variation. TM: Transmembrane.

The protein-coding sequences (.faa file extension) for each species were downloaded from Ensembl v.105^34^ or the NCBI database^35^. In all cases, we kept the longest transcript for each gene. Of all the proteins present in the proteomes, we selected those that had between 2 and 35 transmembrane domains using the software TMHMM v2.0^36^ (Fig. 1). We annotated the protein sequences based on the structural motifs using the program RPS-BLAST v2.13.0 + (with the option -outfmt 11) plus the rpsbproc package^37^ (Fig. 1). We filtered the results based on the *E-value* threshold of 10^–4^ (Fig. 1). To identify the ion channels from our list of proteins, we prepared a file containing the list of ion channel conserved domains based on the Conserved Domain Database (CDD)^38^. Having done that, we intersected it with the results from RPS-BLAST v2.13.0 + followed by rpsbproc. This was done using an *in-house* Perl script to identify the ion channel repertoire for all sampled species (Fig. 1). The result was the list of ion channel protein sequences from each species for the next steps (Supplementary Table S1).

### Homology inference

After having identified the ion channel repertoire from each sampled species, the next step was to infer homologous relationships. To do this, we used the program OMA standalone v2.5^39^. We inferred Orthologous Groups (OGs) containing 1:1 orthologous genes and Hierarchical Orthologous Groups (HOGs) containing the set of genes that have descended from an ancestral gene. OGs were used to perform natural selection analyses by estimating the rate of non-synonymous (d_N_) and synonymous (d_S_) substitutions. In contrast, the HOGs were used to perform gene copy number variation analyses. Both analyses take into account the phylogenetic relationships of the included species. Amino acid sequences were aligned using the FFT-NS-2 strategy of the program MAFFT v7.490^40^. Codon-aligned nucleotide alignments were obtained by employing the amino acid alignments as templates, using the software PAL2NAL^41^.

### Molecular evolution analyses

To test for evidence of positive selection on the cetacean orthologous groups of ion channels, we estimated the ratio of the rate of non-synonymous (d_N_) and synonymous substitutions (d_S_) (ω = d_N_/d_S_) using site models in the program PAML v4.9^42^. For each multiple sequence alignment, we compared models that allow ω to vary among codons (M1a vs. M2a, M7 vs. M8). M2a and M8 models allow a subset of sites to have ω > 1, in contrast to the null models (M1a and M7), which only includes site classes with ω ≤ 1. All nested models were compared using the Likelihood Ratio Test (LRT), and we applied the False Discovery Rate (FDR) as a multiple-test correction^43^.

### Gene turnover rate analyses

To study variation in the gene turnover rate, we used the software CAFE v4.2.1^44^. Using this program, we estimated the rate of evolution (λ) and the direction of the change regarding the size of gene families across different lineages. We implemented two models, (1) in the first model, we estimated one λ value for cetaceans as a total group (crown and stem), and a second λ for all non-cetaceans branches of the tree, and (2) in the second model, we estimated one λ for the stem Cetacea, another λ for the crown Cetacea, and a third λ for all non-cetaceans branches of the tree. The divergence time between species was obtained from the TimeTree 5 database^45^**.**

### Physiological phenotypes associated with the positively selected genes

To understand the physiological phenotypes to which our list of ion channels with the signature of positive selection are associated, we performed an analysis using the mammalian phenotype ontology database^46,47^ as implemented in the Enrichr platform (https://maayanlab.cloud/Enrichr/)^48^. In this analysis, we compared our gene list of positively selected ion channels (query list) against the gene list of the platform (subject list), which has 9767 genes grouped in 4601 phenotypical categories. The platform calculates the adjusted p-values from all raw p-values using the FDR procedure, and we considered only the phenotype categories with adjusted p-values equal to or below 0.01.

### Structural methods

Protein structure homology modeling was performed using the SWISS-MODEL server (https://swissmodel.expasy.org/)^49^. Structural figures were prepared using PyMOL Molecular Graphics System, Version 2.0.6 Schrödinger, LLC. Ligand–protein interaction diagrams were performed with LigPlot^+^ v.2.2^50^.

## Results and discussion

### Ion channel annotation and homology inference

**Figure 2:**
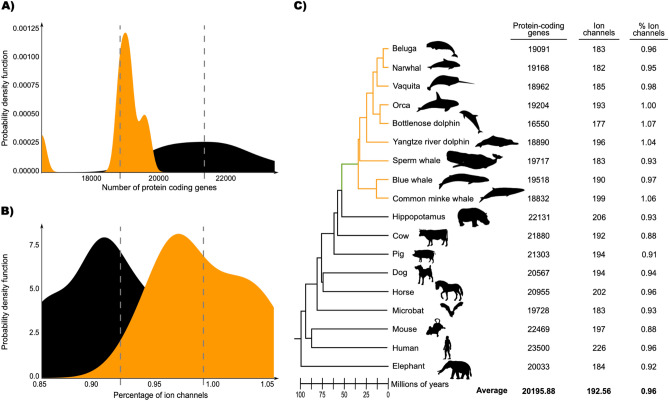
Comparison between averages of cetaceans (orange) versus non-cetaceans (black) in terms of the number of protein-coding genes (**A**) and percentage of ion channels among them (**B**). The Probability Density Functions were inferred through Kernel Density Estimation from the data processed from the genome annotations of the species included in our sampling. (**C**) phylogenetic tree showing the species included in addition to the number of protein-coding genes, ion channels, and the percentage of ion channels. The green branch denotes the stem cetacea, while the orange branches are the crown cetacea. The divergence times were obtained from the Timetree 5 database^45^**.** Phylogenetic relationships were obtained from the literature^54,55^. Silhouette images were downloaded from PhyloPic (CC0 1.0 Universal Public Domain Dedication, https://creativecommons.org/publicdomain/zero/1.0/, http://phylopic.org/).

In this work, we studied the evolution of ion channels in cetaceans. To do this, we designed a bioinformatic pipeline (Fig. 1) to annotate the ion channel repertoire from the proteome of different mammalian species. Based on our analyses**,** we found that cetaceans possess fewer protein-coding genes than non-cetacean mammals (18,845.5 ± 977.29 vs. 21,396.22 ± 1218.75, unpaired one-tailed t-test with d.f. = 14.88; t-statistic = − 4.94 and *p*-value = 9.1e−5) (Fig. 2). This result is consistent with other studies in which a reduction in gene copy number in cetaceans and other groups is associated with evolutionary innovations ^15,17,19–26,51^. We annotated, on average, 192.56 ion channels in the genomes of the species included in our taxonomic sampling, representing 0.96% of the protein-coding genes (Fig. 2). The smallest number of ion channels (177) was obtained for the bottlenose dolphin (*Tursiops truncatus*), while the largest number (226) was in humans (*Homo sapiens*) (Fig. 2). Our results are comparable, to what is reported in the literature: 235 ion channels for humans (*Homo sapiens*) and 231 for the mouse (*Mus musculus*)^27^. According to our results, on average, cetaceans possess a higher proportion of annotated ion channels in their genomes than the non-cetacean mammals (9.95 ± 0.38 vs. 0.92 ± 0.61, unpaired one-tailed t-test with d.f. = 13.587; t-statistic = 2.933 and *p*-value = 0.005) (Fig. 2). Although the literature contains abundant examples of gene loss reported for cetaceans (see references above), there are also examples in which cetaceans expanded their gene repertoire. For instance, Holthaus et al.^52^ report that a subtype of small proline-rich proteins has expanded in cetaceans. Genes related to tumor suppression, cell cycle checkpoint, cell signaling, and proliferation have also expanded their repertoire in cetaceans^1,53^.

Through the homology inference procedure, we obtained 112 orthologous groups of putative ion channels present in all cetacean species. Additionally, 209 hierarchical orthologous groups containing two or more sequences from different species were inferred.

### Ion channels with the signature of positive selection are related to heart, locomotion, vision and neurological physiology

Using site analyses as implemented in PAML, we tested 112 orthologous groups of ion channels in which all cetacean species were present. According to our results, we retrieved 39 orthologous groups that were significant in both comparisons (M1vs M2 and M7 vs. M8). After performing an enrichment analysis of the genes with the signal of positive selection against the mammalian phenotype ontology database^47^, we recovered categories related to phenotypes linked to the group of genes that are well known to have been modified due to the aquatic transition (e.g., circulatory, locomotor, and visual systems, among others). Comparable results have been obtained in previous studies where groups of genes related to specific phenotype are studied^1,53,56^, also in genome-wide studies^7–9,57^.

**Figure 3:**
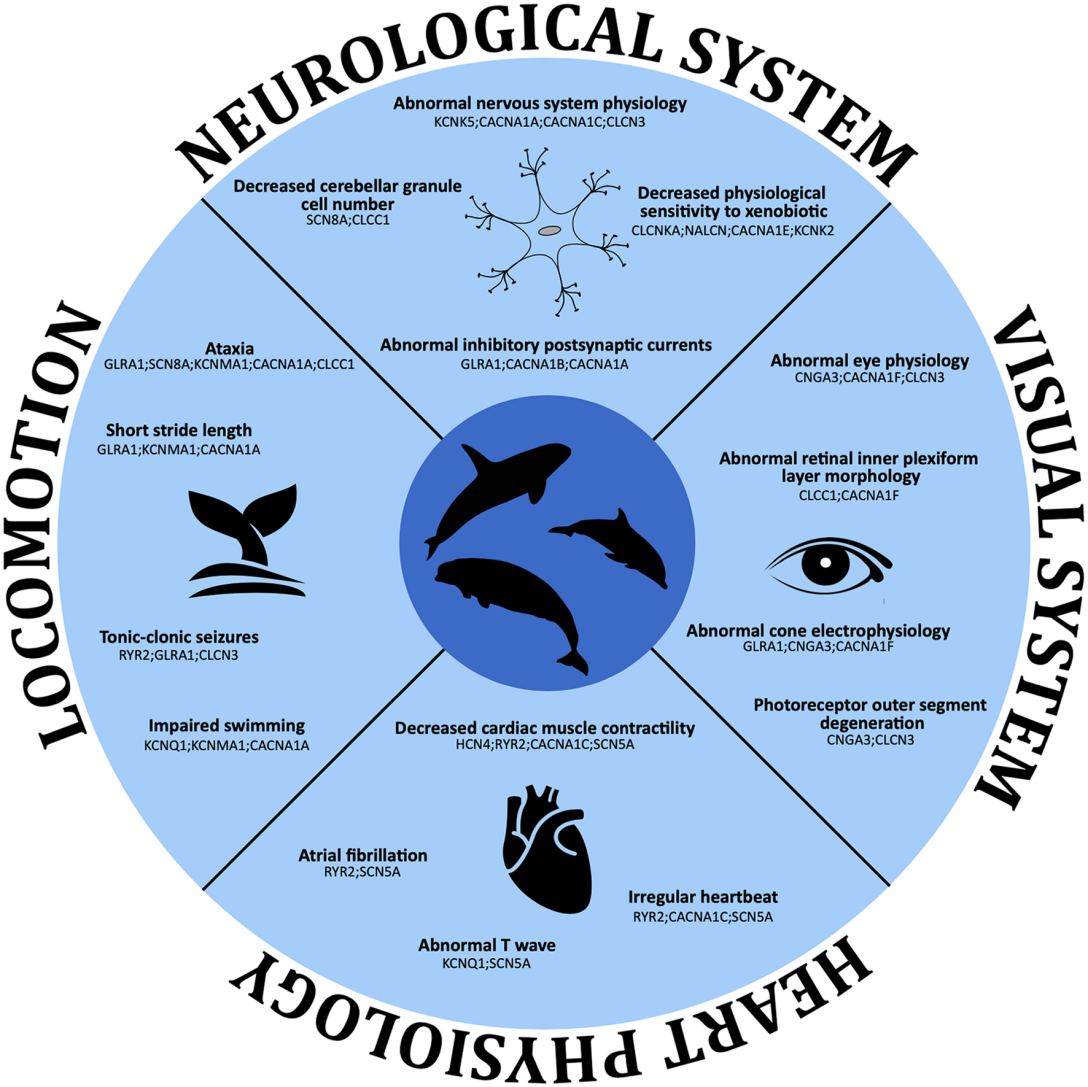
Enriched categories related to heart physiology, locomotion, visual system and neurological system in cetaceans based on the MGI mammalian phenotype ontology database using the Enrichr platform (https://maayanlab.cloud/Enrichr/). For further details see Supplementary Tables S1, S2, S3, S4 and S5.

Our study found a category related to heart physiology. We identified categories such as “decreased cardiac muscle contractility”, “irregular heartbeat”, and “atrial fibrillation”, which align with previous literature on changes in heart function during diving (Fig. 3 and Supplementary Table S2)^58–61^. One of the main challenges for aquatic living is coping with the acute hypoxia that occurs during extended breath-holding. To overcome the physiological effects of deep dives, remarkable adaptations have evolved, including significant decreases in heart rate and redistribution of blood flow to essential aerobic tissues^62^. Although the basic structure of the cetacean heart is similar to that of other mammals, based on our findings we hypothesized the importance of ion channels, among other proteins, in adapting to diving. Thus, besides the well-documented morphological changes in the cetacean heart^63,64^ mostly attributed to diving adaptations, our results show adaptive changes in genes encoding ion channels, which are fundamental for their physiological divergence.

Among the genes associated with the recovered categories related to heart physiology (Fig. 3 and Supplementary Table S2), the Sodium Voltage-Gated Channel Alpha Subunit 5 (SCN5A) gene is the most frequent. The SCN5A gene encodes the alpha subunit of the sodium channel Na_V_1.5, mainly expressed in the heart^65^. This protein mediates the voltage-dependent sodium ion permeability of the cardiac muscle, playing an essential role in regulating cardiac electrophysiological function^66^. In addition, mutations in this ion channel cause a broad range of electrical disorders and structural abnormalities in humans^66–68^. Past studies have also found this ion channel under positive selection in cetaceans^8^.

**Figure 4:**
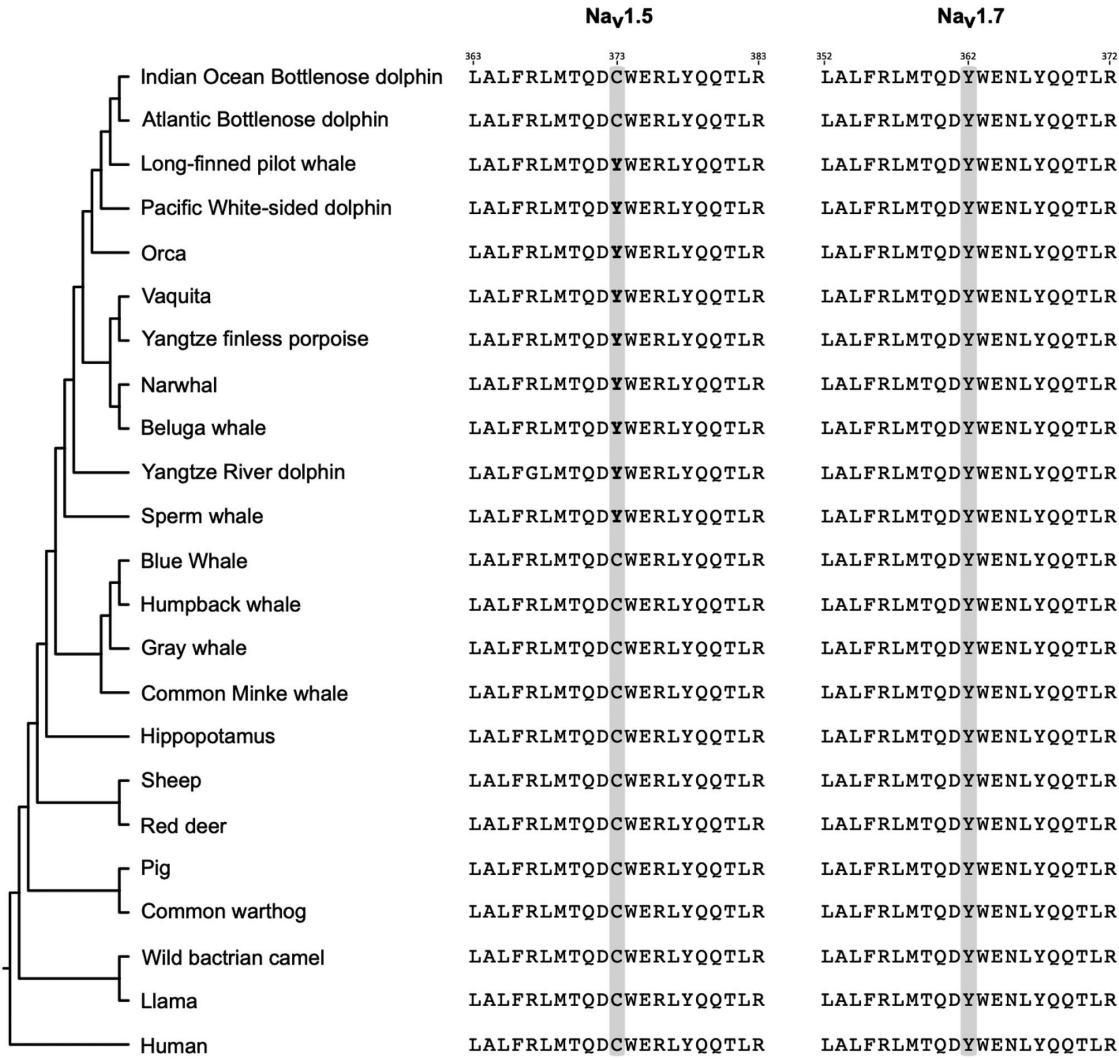
Amino acid alignment of a segment between the transmembrane segments 5 and 6 of the Na_V_1.5 and Na_V_1.7 ion channels containing the amino acid positions responsible for the sensitivity to TTX (shaded in gray). The numbering of the amino acids is with respect to the human sequences (Na_V_1.7, NM_001365536.1; Na_V_1.5, NM_001099404.2). Phylogenetic relationships were obtained from the literature^54,55^. In bold are the amino acids tyrosine (Y) in the Na_V_1.5 ion channels of toothed whales (Odontoceti), which would make them more sensitive to TTX.

**Figure 5:**
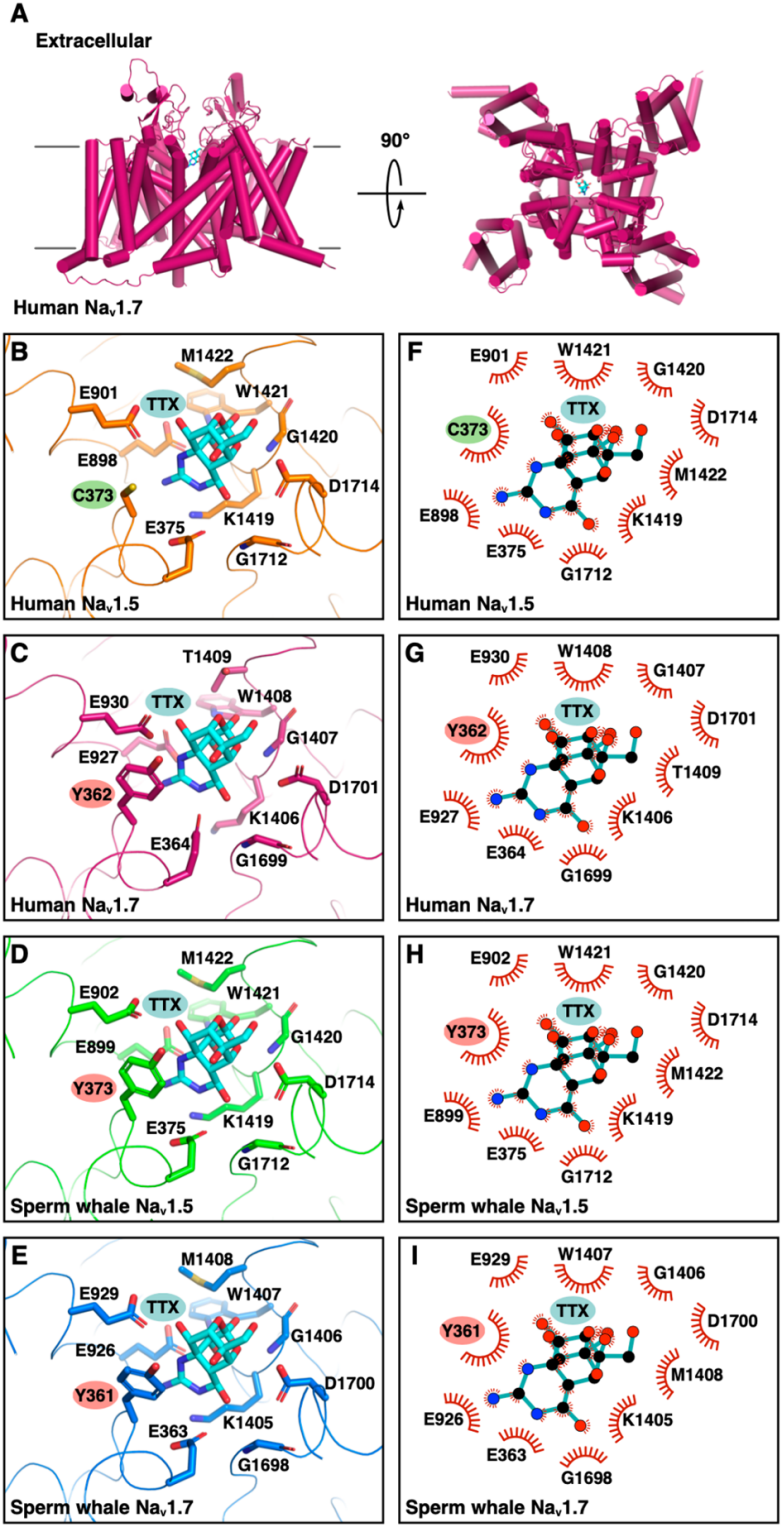
Comparison of the binding site for TTX in human and sperm whale Na_V_1.5 and Na_V_1.7. (**A**) Cartoon representation (alpha-helices shown as cylinders) of human Na_V_1.7 colored magenta, and stick model of TTX with carbon atoms colored cyan (PDB code 6J8I showing only chain B and TTX). (**B**–**E**) Ribbon representation of the TTX binding site on the indicated sodium channels, and stick representation of sodium channel TTX binding site amino acid residues with carbon atoms colored orange (**B**; human Na_V_1.5; PDB code 6LQA), magenta (**C**; human Na_V_1.7; PDB code 6J8I), green (**D**; sperm whale Na_V_1.5; model generated by SWISS-MODEL), or blue (**E**; sperm whale Na_V_1.7; model generated by SWISS-MODEL), and of TTX with carbon atoms colored cyan. Highlighted are the positions of C373 in human Na_V_1.5, of Y362 in human Na_V_1.7 which is critical for binding via a π-cation interaction with the 1,2,3-guanidinium group of TTX, and of Y373 and Y361 of sperm whale Na_V_1.5 and Na_V_1.7, respectively, predicted to interact with TTX via a similar p-cation interaction. The carbonyl and amino groups of glycine residues are depicted for better visualization. (**F**–**I**) Two-dimensional, schematic representation of the position of TTX shown in B-E using LigPlot^+^.

Although the Na_V_1.5 and its paralog, the Sodium Voltage-Gated Channel Alpha Subunit 9 (Na_V_1.7) sodium channel have similar selectivity filters^68,69^, the Na_V_1.5 channel has an affinity about two orders of magnitude lower for tetrodotoxin (TTX)^70,71^, a sodium channel blocker that is found in a variety of marine animals (e.g., pufferfish, horseshoe crabs, blue-ringed octopus, gastropods, starfish, among others)^72^. This difference is due to a single amino acid substitution, where the Na_V_1.5 channel has a cysteine at position 373 instead of a tyrosine (Fig. 4). As expected, all Na_V_1.7 channels of cetaceans possess a tyrosine amino acid at a structurally related position, Y362 in human Na_V_1.7 (Fig. 4), making it susceptible to TTX blockage. Interestingly, besides the bottlenose dolphins, the Na_V_1.5 channel of the toothed whales (Odontoceti) species included in our study possesses a tyrosine instead of cysteine (Fig. 4), making it potentially sensitive to TTX. Furthermore, three-dimensional protein structure modeling of sperm whale Na_V_1.5 and Na_V_1.7 shows a conserved spatial arrangement of amino acid residues that are important for TTX binding on human Na_V_1.7, including Y362 that establishes a π-cation interaction with the guanidinium group of TTX, an interaction that is not possible by C373 of human Na_V_1.5 (Fig. 5). To strengthen our conclusions, we retrieved additional Na_V_1.5 sequences from toothed whales to see if they also have tyrosine instead of cysteine. In our new searches, we recovered the Na_V_1.5 ion channel of the long-finned pilot whale (*Globicephala melas*), Pacific white-sided dolphin (*Lagenorhynchus obliquidens*) and Yangtze finless porpoise (*Neophocaena asiaeorientalis*). We found a tyrosine residue in all cases, providing further support to our results (Fig. 4). We also found a different species of the genus *Tursiops*, the Indo-Pacific bottlenose dolphin (*Tursiops aduncus*), and following the trend we are describing, it has a cysteine residue (Fig. 4). Finally, to be sure that this cysteine to tyrosine replacement only occurred in toothed whales, we also retrieved additional Na_V_1.5 sequences from baleen whales (Mysticeti). We added two more species, the humpback whale (*Megaptera novaeangliae*) and the gray whale (*Eschrichtius robustus*). Both species have a cysteine residue, confirming our initial conclusion (Fig. 4). Thus, the amino acid substitution from cysteine to tyrosine occurred in the last common ancestor of toothed whales (Odontoceti), which lived between 34 and 33 million years ago^45^. However, in the last common ancestor of the genus *Tursiops*, it was reversed (Fig. 4). This is interesting because documentarists have been able to record dolphins "playing" with pufferfish, with the threat of being exposed to TTX that the pufferfish releases when it feels threatened. However, since the toxin is released into the water, it seems to be not in lethal doses, and it has been proposed it could cause a trance-like state. This could be possible because pufferfish mainly accumulate TTX in the skin^73^, especially when they are young^74^, an organ in direct contact with the mouth of the dolphin. In fact, it has been observed that dolphins behave differently after "playing" with the pufferfish. Therefore, this behavior could be related to the reversal in the amino acid substitution, which makes it plausible that the Na_V_1.5 ion channel of the species of the genus *Tursiops* has a lower affinity for TTX, like all mammals. Thus, besides having mutations associated with human channelopathies, the Na_V_1.5 channel of a subset of cetaceans, toothed whales (Odontoceti), is predicted to have a higher affinity to TTX than other mammals. Further lab experiments are the next logical step to reveal the functional divergence of the Na_V_1.5 in this particular group of mammals.

The Ryanodine Receptor 2 (RYR2) gene, the second most frequent gene and encodes for a protein that is one of the components of the largest ion channel known to date. It is mainly expressed in the heart^65^, and its primary function is controlling Ca^+2^ release from the sarcoplasmic reticulum throughout the cardiac cycle^75^. Mutations in this channel have been associated with Arrhythmogenic Right Ventricular Dysplasia, familial, 2 (ARVD2), Ventricular tachycardia, catecholaminergic polymorphic, 1 (CPVT1), Ventricular arrhythmias due to cardiac ryanodine receptor calcium release deficiency syndrome (VACRDS), among others^76^.

We also retrieved categories related to locomotion (Fig. 3 and Supplementary Table S3), one of the phenotypes significantly modified in cetaceans, with profound changes in the body plan due to the aquatic transition. Unlike terrestrial mammals, cetaceans have elongated bodies and absent hindlimbs. The forelimb was modified into a flipper, and vertical movements of the tail accomplished locomotion. Moreover, moving into the water column imposes a highly energetic cost requiring a greater force than moving into the terrestrial environment, which translates into a need for stronger muscle contraction^12^. In stronger muscle contraction, ion channels play a fundamental role by initiating the influx of ions, including sodium transport, into the cells. This result agrees with Sun et al.^8^, which reported the signature of positive selection in genes related to motor activity and muscle contraction in cetaceans. The two most frequently mentioned ion channels are the Calcium Voltage-Gated Channel Subunit Alpha1 A (CACNA1A) and Glycine Receptor Alpha 1 (GLRA1). Thus, like the heart case previously described, given that marine mammal muscle architecture is similar to most other mammals^77^, the fact that we recovered several ion channels related to locomotion highlights their fundamental role in physiological divergence.

The senses of cetaceans have radically changed in association with aquatic living^78^. The cetacean visual system has been modified to meet the challenges of the aquatic way of life, in which ion channels seem to be an important factor (Fig. 3 and Supplementary Table S4). Unlike many terrestrial mammals, cetaceans have eyes positioned laterally on their heads, which provide a panoramic view of their surroundings. This lateral placement is crucial for wide-range detection of both predators and preys in their vast aquatic habitat^79^. Although cetacean eyesight is generally considered less acute than that of terrestrial mammals, it includes specific adaptations for underwater vision. For example, a key adaptation is the higher density of rod cells in their retinas, implying enhanced vision in low-light conditions prevalent in deeper waters^80^. Also, cetaceans lack a fovea, a slight depression within the retina, a region associated with visual acuity in other mammals^81^. This absence suggests an evolutionary trade-off, where the demands of aquatic vision have shaped a different visual acuity strategy. Furthemore, the evolutionary history of cetacean opsin genes, responsible for light detection, reflects an adaptation to the marine light environment^79,82^.

We also identified a category related to the neurological system (Fig. 3). This category encompasses all aspects of phenotypic changes within the nervous system attributed to the diverse sensory adaptations now thoroughly documented as originating from the transition to aquatic life^78,83–85^. These phenotypic changes include modifications in brain morphology, sensory processing, and motor control to support their aquatic lifestyle^77^. Accordingly, we retrieved categories associated with abnormal nervous system physiology, brain morphology, and excitatory postsynaptic potential, among others (Fig. 3 and Supplementary Table S5). The most frequent genes in this category are CACNA1A and SCN8A. Associated with this category, we also retrieved ion channels related to hearing, although not among the most significantly enriched categories. We identified ion channels associated with ear and otolith morphology anomalies, among other findings. Accompanying the specialized hearing capabilities in this mammalian group, neuroanatomical alterations affecting the inner ear and cranial nerves have been documented^78^.

### Accelerated gene turnover rate in cetaceans

The availability of whole-genome sequences has revealed that variation in gene copy number is abundant in nature^86^ and related to the origin of phenotypic diversity. For example, a survey of more than 9000 gene families in primates suggested that humans possess faster gene turnover than other mammals^87^. In this study, the authors found several expansions (e.g., centaurin gamma gene family) in the lineage leading to humans that are related to the unique attributes of our species (e.g., enlarged brains)^87^, establishing a link between copy number variation and evolutionary innovations.

**Figure 6:**
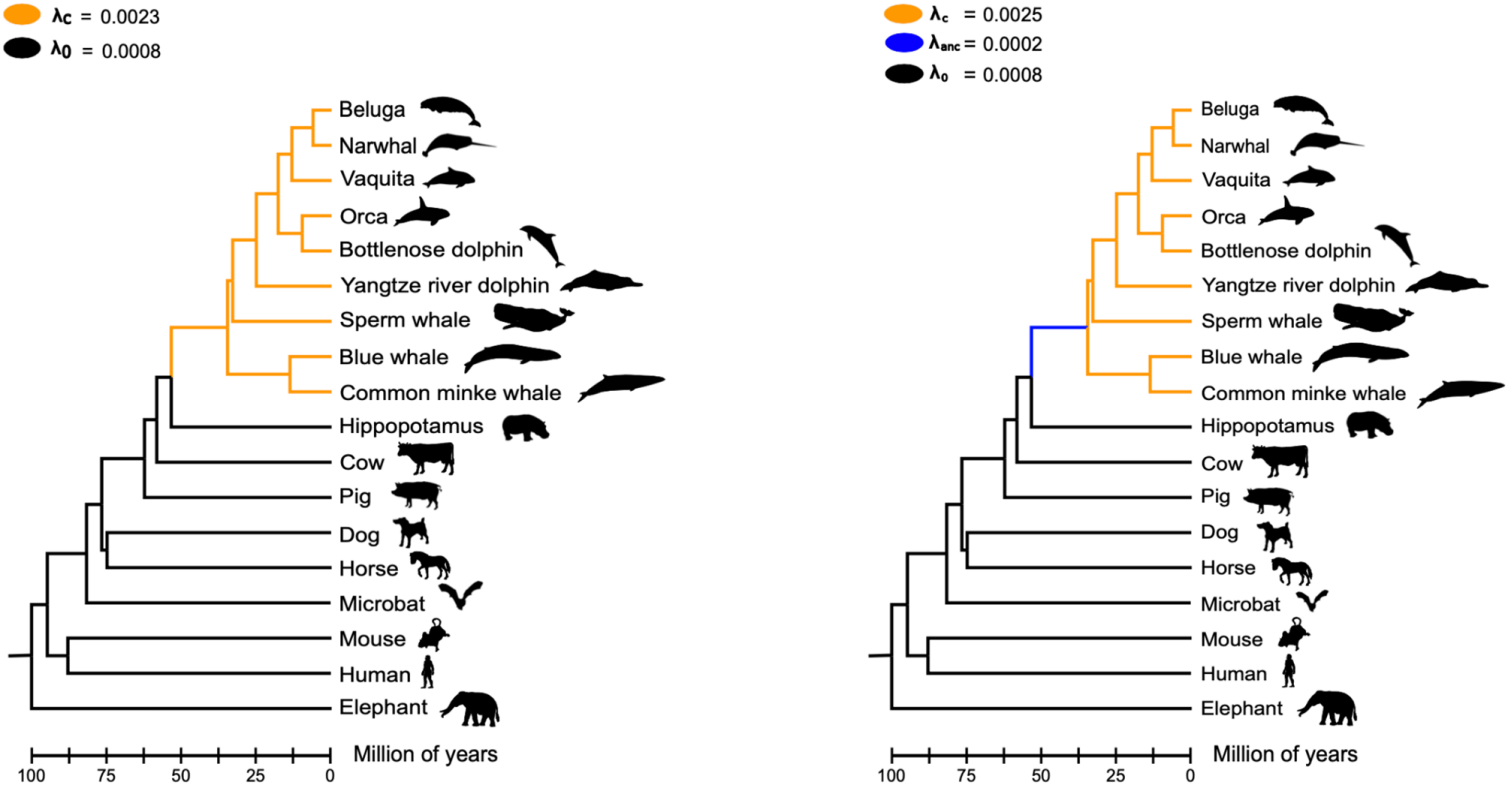
Gene turnover rates of ion channels. The first model (left panel) estimated the rate of evolution (λ) of ion channels for cetaceans as a total group (orange branches) and for non-cetacean mammals (black branches). Under this model, the λ value for cetaceans is more than two times faster than non-cetacean mammals. The second model (right panel) estimated λ values for the last common ancestor of cetaceans (blue branch), for the crown group cetacea (orange branches), and for non-cetacean mammals (black branches).

In our results, we also found variation in the gene turnover rate (Fig. 6). Our models estimating different λ parameters for cetaceans, as a total group and non-cetacean mammals, showed that the first group possesses a rate of evolution (λ_C_ = 0.0023) 2.87 times faster compared to non-cetacean mammals (λ_o_ = 0.0008; Fig. 6). In the second model the estimated λ parameter for the crown group cetacea (λ_C_ = 0.0025) was 3.12 times faster in comparison to non-cetacean mammals (λ_0_ = 0.0008) and 12.5 times faster than the value estimated for the last common ancestor of cetaceans (λ_anc_ = 0.0002) (Fig. 6), suggesting that most of the variation in gene copy number occurred during the radiation of the group. Similar results were obtained when tumor suppressor genes were analyzed^1^.

**Figure 7:**
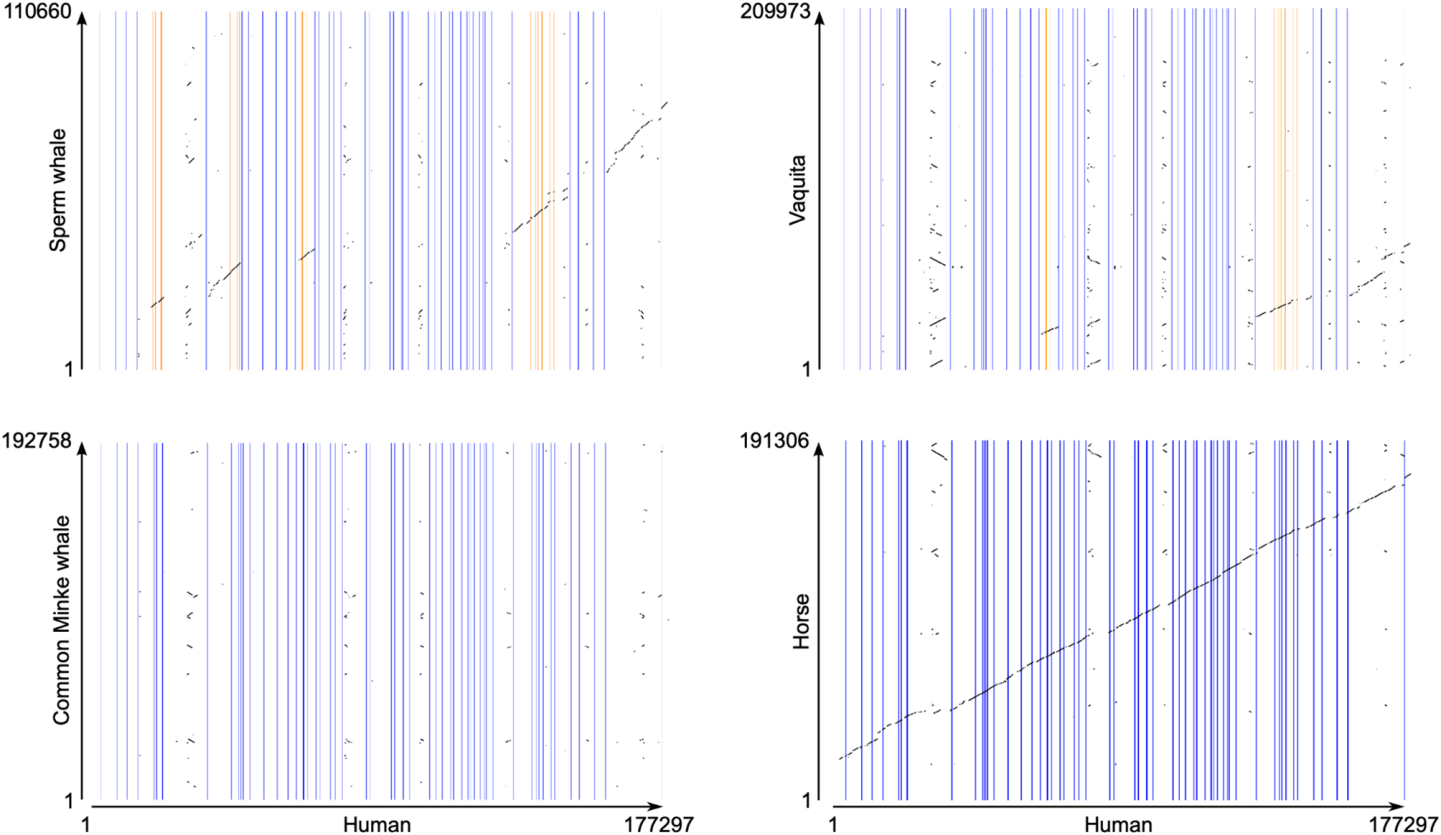
Pairwise dot-plot comparison of the genomic region containing the polycystic kidney disease protein 1-like 1 (PKD1L1) gene of the human (*Homo sapiens*) with the corresponding region in the sperm whale (*Physeter macrocephalus*), common minke whale (*Balaenoptera acutorostrata*), vaquita (*Phocoena sinus*), and horse (*Equus caballus*). Vertical lines denote exons, and regions in between are introns. Orange vertical lines indicate exons that are still present in the species that lost the PKD1L1 gene.

According to our estimates, the number of copies of ion channel genes in cetaceans varies between zero and 18. We found four hierarchical orthologous groups in which all cetacean species have no copies (ASIC5, CLDND1, KCNMB3, and PKD1L1). In these cases, we double-checked the information and found different situations. In the case of the acid-sensing ion channel 5 (ASIC5), we found it in the FASTA file containing all protein-coding genes of baleen whales (Mysticeti), but it was predicted to have less than two transmembrane segments, so it was not included in the next step in our pipeline. A similar situation occurred for the calcium-activated potassium channel subunit beta-3 (KCNMB3) and Claudin domain containing 1 (CLDND1) genes. The polycystic kidney disease protein 1-like 1 (PKD1L1) gene was only found in Orca, but it was not predicted to have any transmembrane segment. We also noticed that the PKD1L1 sequence associated with the accession number (XP_033267232.1) was removed from NCBI. According to ENSEMBL, no cetacean species was predicted to have an ortholog of the human PKD1L1 gene. Further, a comparison of the genomic region of the human (*Homo sapiens*), which possesses the PKD1L1 gene, with the corresponding chromosomal region in the sperm whale (*Physeter macrocephalus*), common minke whale (*Balaenoptera acutorostrata*), and vaquita (*Phocoena sinus*) suggests that the PKD1L1 gene is not present in the cetacean lineage (Fig. 7). It is worth noting that traces of the PKD1L1 gene are present in the sperm whale and vaquita genomes (Fig. 7). In Fig. 7 we also included the comparison with the horse (*Equus caballus)*, a species that share a common ancestor with humans at the same age as cetaceans, to show the conservation pattern of the chromosomal region harboring the PKD1L1 gene (Fig. 7). So, it is highly probable that this gene is not present in the cetacean genome. This result agrees with the study of Turakhia et al.^88^; however, they also show that gene loss is not a cetacean-specific evolutionary event, as they did not find the PKD1L1 gene in other cetartiodactyla species (e.g., alpaca, Bactrian camel, goat, sheep, Tibetan antelope, and cow)^88^. In agreement, the cow and pig genome lack the PKD1L1 hierarchical orthologous group. Thus, this result suggests that the deletion of the PKD1L1 gene occurred in the last common ancestor of cetartiodactyla. PKD1L1 is a member of the TRP gene family, mainly expressed in the testis and heart^89,90^. It also has functions related to establishing left–right asymmetry in positioning and patterning internal organs and associated vasculature by forming heteromeric channels with PKD2, functioning as sensors of the nodal flow^91–95^. However, this gene loss does not translate into functional consequences in cetaceans and even-toed ungulates, probably due to a certain degree of redundancy that could serve as a backup with functionally overlapping family members^96–98^.

The hierarchical orthologous group that contained the highest number of copies corresponded to the transmembrane protein 37 (TMEM37), a gamma subunit of voltage-gated calcium channels, with 18 copies. In the case of the non-cetacean mammals in our sampling, this hierarchical orthologous group has nine copies, suggesting that the 18 copies represent a cetacean-specific expansion. After that hierarchical orthologous group with 18 gene copies, there is a group with 12 copies (P2RX4), another with 11 copies (KCNK13) and two groups with 10 copies (CLCN4 and KCNH4). In all cases, the non-cetacean mammals in our sampling have fewer copies than cetaceans, six for P2RX4 and nine for KCNK13, CLCN4 and KCNH4.

A more detailed assessment of the hierarchical orthologous groups provides a better panorama of the evolutionary trend in which cetacean species possess fewer ion channels than the non-cetacean mammals in our sampling. Our analysis found that out of the 209 hierarchical orthologous groups inferred, cetaceans possess more gene copies for 51 groups. On the other hand, in 94 hierarchical orthologous groups, both groups have the same number of gene copies, and, in 64 hierarchical orthologous groups, non-cetacean mammals possess more copies than cetaceans.

## Conclusions

In this work, we designed a bioinformatic pipeline that identifies the entire repertoire of ion channels of any species. In our case, we used it to study the evolution of ion channels in cetaceans, a mammalian group that, due to the conquest of the aquatic environment, has extensively modified physiological axes in which ion channels play a significant role. Our results indicate that cetaceans have on average, fewer protein-coding genes and a higher percentage of annotated ion channels than non-cetacean mammals. Furthermore, most of the genes with the signal of positive selection are related to heart, locomotion, visual and neurological phenotypes, consistent with previous studies. The Na_V_1.5 channel of mammals is about two orders of magnitude less sensitive to TTX than NaV1.7. This difference is due to a cysteine residue instead of a tyrosine in a specific position. However, our work shows that most species of toothed whales (Odontoceti) possess a tyrosine amino acid residue in that particular position of the Na_V_1.5 channel, making them potentially sensitive to TTX, similar to Na_V_1.7. However, it is important to recall that the effect of an amino acid replacement depends on the genetic background. So, future studies should be directed to evaluate the biochemical/biophysical performance of the Na_V_1.5 channel of toothed whales, especially their sensitivity to TTX. Finally, the natural experiment that cetaceans represent in the Tree of Life provides an excellent model to advance our understanding of the genetic bases of phenotypic diversity. The number of genomes that exist today and those that are yet to come will make the field of evolutionary genomics a significant contributor to different disciplines in biology and medicine.

### Supplementary Information

Supplementary Table S1.

Supplementary Tables.

## Data Availability

Data and supplementary material are available online at https://github.com/opazolab/Cetacean_ion_channels
